# The Effects of Boko Haram Terrorism on Adolescents' and Young Adults' Psychosocial Adjustment in the Northeast of Nigeria

**DOI:** 10.1177/13634615261436997

**Published:** 2026-05-13

**Authors:** Wasiu Olorunlambe, Andreas Jud

**Affiliations:** 1 Department of Child and Adolescent Psychiatry/Psychotherapy, 9189 Ulm University, Ulm, Germany; 2 Competence Centre Child Protection, Ulm, Germany; 3 207470 School of Social Work, Zurich University of Applied Sciences ZHAW, Zurich, Switzerland

**Keywords:** Boko Haram terrorism, mental health, psychosocial adjustment, adolescents, Northeast, Nigeria

## Abstract

Children are a particularly vulnerable group and, in northeast Nigeria, they are severely affected by Boko Haram terrorism. Although previous studies have looked at the effects of terrorism on children’s mental health, there is a lack of peer-reviewed studies on the effects of terrorism on adolescents’ psychosocial adjustment. This study addresses this gap with data on the consequences of Boko Haram terrorism in the northeast of Nigeria. A cross-sectional research design was adopted through systematic sampling. In total, 391 children who were directly affected by Boko Haram terrorism were surveyed. A majority of the sample was male (60.4%). Respondent age ranged from 11 to 23 years (*M* = 18.00, *SD* = 2.83). Analyses revealed that 291 (74.42%) of the respondents reported severe traumatic exposure—a markedly increased percentage compared with prevalence rates in countries without conflict. The majority of respondents (70.4%) reported clinically significant symptoms of post-traumatic stress disorder (PTSD). PTSD regularly co-occurred with other mental health and behavioral symptoms: depression, anxiety, and aggression. Terrorism-induced distress had an adverse effect on psychosocial adjustment (*R*^2 ^= .364, *F*(6, 385) *p* < .01). For example, it was found that children were restricted in their social life. Boko Haram terrorism has a devastating impact on adolescents’ mental health; most victims presented clinically relevant symptoms of PTSD. The victimized respondents were affected in their psychosocial adjustment to daily life and their educational development. Their vulnerability increases the risk of becoming potential recruits for terrorists. Supporting the victims would thus not only reduce their suffering but also decrease the likelihood of an ongoing cycle of violence.

Globally, statistics show that 6,722 terror-related incidents were reported in 2019, leading to the death of 13,822 civilians, physical injuries in 14,542 people, and the abduction of 4,664 individuals (National Consortium for the Study of Terrorism and Responses to Terrorism, 2020). These terrorist-related deaths occurred in five countries: Afghanistan, Yemen, Iraq, India, and Nigeria. Terrorism remains a major security problem in Nigeria (Albert, 2021). The phrase Boko Haram simply means “Western education is forbidden” (Adesoji, 2010). The activities of the Boko Haram terrorist group began in 2002 in Maiduguri, Borno State, northeastern Nigeria, but became known to the public in 2009 following the killing of its founder, Mohammed Yusuf, by the Nigerian Police on 30 July 2009 (Albert, 2021). On 14 April 2014, the group attracted global attention and condemnation when an estimated 276 adolescent girls were kidnapped at Chibok Secondary School, Borno State. Some of the girls were married off, while the whereabouts of others remain unknown (BBC News, 2021; Ibrahim & Mukhtar, 2017).

Northeastern Nigeria has become increasingly dangerous for children, with a record number being directly exposed to Boko Haram terrorism (Wessells & Kostelny, 2021). Reports revealed that, between December 2020 and June 2021, about 936 school children were abducted by Boko Haram terrorists across northeast Nigeria (Tolu-Kolawole & Altine, 2021). In addition, no fewer than 300,000 children have reportedly lost their lives through the activities of Boko Haram terrorists over the past 12 years. Similarly, a UNICEF report indicates that 1.9 million civilians are internally displaced by the group, approximately 60% of them children (UNICEF, 2020), and 20,000 children have been separated from their families (Protection Fact Sheet on Suicide Attacks, 2016). In addition, about 70,000 adolescent girls have been sexually assaulted by Boko Haram members since 2009, a large proportion of whom have been forced into marriage (Ali et al., 2016), which contributes to Nigeria having the highest number of child marriages in Africa with 23 million girls married before the age of 18 years (United Nations Development Programme (UNDP), 2019).

Although a growing body of research suggests that children and adolescents are severely affected by armed conflict (Saraiya et al., 2013; Strelitz et al., 2018), few studies have explicitly focused on the effects of terrorism on children. Exposure to terror attacks may undermine children's psychosocial functioning, educational trajectories, social/healthy living, and normative development across the lifespan (Bharadwaj et al., 2021; Eisenberg & Silver, 2011; Terwase et al., 2015). In northeastern Nigeria, children caught up in Boko Haram terrorism also add to the burden of *Almajiri* (displaced/destitute children) who roam the region's major cities. In general, Boko Haram terrorism in Nigeria poses a grave threat to the realization of United Nations Sustainable Development Goal 16, which is to eradicate all forms of violence against children by 2030 and promote just, peaceful, and inclusive societies (Brown et al., 2016). Victims of terrorism, particularly children and adolescents, experience consequences to their psychosocial adjustment; namely, their capability to deal with basic daily routines, such as self-care, social relations, as well as school and recreational activities (Skodol, 2018). Psychosocial adjustment is associated with social interactions, school functions, and personal well-being, and poor psychosocial adjustment in adolescence is associated with a poor mental outcome in adulthood (Tayfur et al., 2022). Life events are significant occurrences that disrupt individuals’ lives and often require psychosocial adaption (Betancourt et al., 2010). In the context of Boko Haram, children affected by terrorism experience adverse life events that can significantly shape their life trajectories and may result in lifelong mental health consequences if not addressed early. Many of these children have been exposed to multiple, prolonged and overlapping adversities, such as witnessing bombings, losing or seeing their parents severely injured, being abducted from schools, and being forcibly recruited or used in suicide attacks (O’Connor et al., 2021).

The nexus between exposure to terrorism and children's mental health is an area of scientific inquiry that has received little research attention. Previous studies on this subject focused primarily on post-traumatic stress disorder (PTSD) and had a narrow geographical scope: the Western world and the Middle East (Fekih-Romdhane et al., 2017; Muro, 2015; Scrimin et al., 2006; Shaar, 2013; Tol et al., 2013; Turrini et al., 2017; Watkins, 2017). There is a lack of empirical evidence on the topic in non-Western countries, particularly in sub-Saharan Africa (Borba et al., 2016; García-Vera et al., 2014; Jordans et al., 2016), and on psychosocial adjustment in the aftermath of terrorism. This study attempts to fill this knowledge gap by providing a nuanced understanding of the effects of Boko Haram on the psychosocial adjustment of children in northeastern Nigeria.

Empirical research on the psychopathological outcomes of terrorist attacks has grown steadily, particularly since 11 September 2001 (9/11), when the World Trade Centre in the United States was attacked (Prieto et. al., 2021). Subsequently, there has been a substantial increase in scientific research on the impact of terrorist attacks on children (Eruyar et al., 2018; Jordans et al., 2018; Miller & Jordans, 2016; Rees et al., 2015). For instance, a meta-analysis involving 43 studies reported that 15.9% of children who had been exposed to conflict were subsequently diagnosed with PTSD (Alisic et al., 2014). In addition, existing studies have found the co-occurrence of two or more pathological disorders among the victims of terrorist attacks; Neria et al., 2011; Perlman et al., 2011). Studies have further established links between decreased mental health and lowered psychosocial functioning in children affected by terrorist conflicts (Betancourt et al., 20100, 2013; Neria et al., 2011; Nordanger et al., 2013; Pereda, 2013; Santiago et al., 2013; Slone & Mann, 2016; Trickey et al., 2012). Recent evidence demonstrated a 10%–30% increase in PTSD among children following a terror incident (García-Vera et al., 2021). With regard to gender differences, empirical evidence suggests that PTSD is more prevalent in girls than in boys after terror-related incidents (Kilpatrick et al., 2013; Pineles et al., 2017 Sever et al., 2008).

The deleterious effects of terrorism on children are not restricted to mental health alone. One in five children living in camps in the areas affected by Boko Haram carnage suffer chronic malnutrition (National Population Commission (NPC), 2014). Children's suffering is further worsened by an inadequate and overstretched child protection system to cater for their health, psychosocial, and humanitarian needs (Pfefferbaum et al., 2012; Stene Dyb, 2015). Globally, meta-analyses have shown a consistent pattern of child responses to terrorism, which include emotional numbness, PTSD, acute stress disorder, anxiety, separation anxiety, sleep difficulties, and depression (Neria et al., 2011; Perlman et al., 2011).

However, there are *variations* in children's reactions to terrorist attacks across the world. In the United States, for example, two-thirds (60.5%) of children expressed intense fear following the 9/11 terrorist attack (Hock et al., 2004). By contrast, terrorist-exposed Israeli adolescents experienced increased drug abuse and anti-social behavior (Pat-Horenczyk et al., 2007). Boko Haram's attacks on educational institutions are similar in terms of ideological motive to the Beslan terrorist attack in Russia, in which 186 school children were killed (Solomon & Dekel, 2008), and the December 2014 attack in Peshawar, Pakistan, in which a scores of high school students were killed (Kalim & Janjua, 2019). Unlike the isolated terrorist incidents that took place in France, Belgium, Spain, and Norway, children living in the northeast of Nigeria experience ongoing psychosocial trauma.

In addition, because of the Boko Haram onslaught, children living in the northeast of Nigeria have long been exposed to multiple adversities and an increased risk of preventable diseases, such as polio and hepatitis (Etsano et al., 2015). Moreover, the near-collapse of economic activity and closure of health facilities because of Boko Haram terrorism have made it extremely difficult for parents and caregivers to feed their children and register them for polio vaccinations (Ekhator-Mobayode & Asfaw, 2018). In 2013, for instance, 50% of polio outbreaks occurred in the northeast region (Etsano et al., 2015). In addition, children living in the northeast of Nigeria were also exposed to recurrent farmer–herdsmen clashes that plagued the region for several years before the emergence of Boko Haram terrorism (Goke, 2018).

## Aim and Objectives

The aim of this study is three-fold. First, to examine the effects of life-change events on the psychosocial adjustment of terrorism-exposed children and young adults**.** Second, to explore the association between traumatic exposure/ stressors and the severity of PTSD symptoms. Third, to determine the comorbidity of PTSD with other mental health illnesses among the victims.

## Methodology

A cross-sectional research design was adopted. The study was conducted among children and young adults in the terrorism-endemic northeast of Nigeria, a multiethnic and culturally diverse country with an estimated population of 206,739,928 people (Population Stat, 2020). Northern Nigeria, which is home to the Hausa, Fulani, and Kanuri communities, remains the most vulnerable and educationally disadvantaged region, with a long history of radical ideological movements (Dan-Azumi, 2025). In 2018, Nigeria was ranked 158 of 189 countries in terms of the Human Development Index (HDI) (UNDP, 2018), with the northern region having an HDI below the national average.

### Study Population and Participant Recruitment

This study included 391 adolescents and young adults aged 11–23 years (*M* = 18.00, *SD* = 2.83) who have been directly affected by Boko Haram terrorism ( loss of parents/relatives, displacement, destruction of homes, hospitals, and schools). The study was conducted in Fufore and Malkohi, internally displaced persons (IDP) camps in Adamawa State, as well as in Madinatu and Shuwari IDP camps in Maiduguri Borno State. The rationale for conducting the study in IDP camps was to ensure access to the vulnerable population while guaranteeing the security of both the research teams and the participants.

To achieve a systematic and representative sample, IDP camps were stratified into neighborhood clusters of households using the random sampling technique. Using the random-route approach, the first household was selected through the systematic sampling technique and the process continued along a route, with standard intervals of five households between the first and the next sampling unit (Reichel & Morales, 2017).

Within the selected households all adolescents and young adults aged between 10 and 23 years, both male and female, were eligible, and the participants were selected using a random number generator. One eligible respondent per household was assessed. Participants aged 10–23 years were included to capture a continuous developmental period during which core emotional, cognitive, and psychosocial processes mature. A further rationale for including both adolescents and emerging adults in this study was that developmental transitions under conditions of conflict and chronic stress are often disrupted and delayed, rendering chronological age differences less distinct (Becht et al., 2021; Ferguson et al., 2021).

#### Inclusion Criteria

Adolescents between 10 and 23 years of age who had been directly exposed to terrorist attacks or whose parents/caregivers or relatives had been killed by Boko Haram terrorists were included in this study (in line with criterion A1 of PTSD in the Diagnostic and Statistical Manual of Mental Disorders, fifth edition (DSM-5)).

#### Exclusion Criteria

The following exclusion criteria were used: (a) respondents who stay outside IDP camps; (b) adolescents suffering from an intellectual disability or psychotic disorder were excluded from the study to ensure reliable and valid data, protect respondents’ safety, and minimize the risk of psychological harm; and (c) a refusal to give assent.

### Measures

The following standardized instruments were included in the survey. These English-language questionnaires were translated into Hausa and back-translated into English by bilingual translators familiar with the subject matter, using the World Health Organization (WHO) protocol.

1. The Psychosocial Adjustment Scale (Ravens-Sieberer & the KIDSCREEN Group Europe, 2006). This 31-item scale assesses the health-related quality-of-life and well-being of adolescents aged 10–19 years, consistent with the WHO definition of adolescence. Responses were rated using a five-point Likert scale, ranging from *never* to *always* or from *not at all* to *extremely*. The scale has demonstrated high internal consistency (Cronbach's α = .82) and test–retest reliability (*r* = .73) (Aymerich et al., 2005). It has also been validated in Uganda, which is similar to the Nigerian context (Masquillier et al., 2012).
2. The Post-traumatic Stress Disorder Checklist for DSM-5. This comprises 20 questions that evaluate the symptoms of PTSD, as contained in the DSM-5 (Ashbaugh et al., 2016). Participants responded to the checklist by indicating the extent to which each of the symptoms happened to them in the past month. Participants’ responses were measured using a five-point Likert scale, anchored from 0 (‘not at all’) to 4 (‘extremely’). This checklist helped to determine an overall score of severity (by summing up the scores obtained for the 20 questions). Previous internal consistency was measured as Cronbach's α = .95. The scale demonstrated good internal consistency in this study at Cronbach's α = .86. This scale was, however, used only to determine symptoms severity of PTSD. We could not conduct diagnostic interviews for the participants because of the high level of insecurity during fieldwork. The PTSD Checklist for DSM-5 used in this study was a symptoms score/predictive scale and has demonstrated high predictive accuracy, sensitivity (75%–100%) and specificity (67%–94%) (Jiang, 2019; G. Li et al., 2018). The PTSD Checklist has been validated in Zimbabwe, which is similar to the Nigerian context (Verhey et al., 2018). The cut-off score of 33 used in this study was to assess symptom diagnosis and was consistent witht Wortmann et al. (2016).
3. The Peritraumatic Distress Inventory (Brunet et al., 2001). This tool consists of 13 questions and evaluates reactions to emotional stress that occurred before and after exposure to a traumatic situation. Items are measured using a five-point Likert scale ranging from 0 (*not at all true*) to 4 (*extremely true*). The overall score is the sum of the scores for all questions. A score >15 depicts high distress. The test–retest reliability coefficient was Cronbach's α = .87.
4. The Adolescent Life-Change Events Scale (Yeaworth et al., 1992). This tool comprises 36 items that measure five dimensions: health, social life, family, school and, emotional life. The scale was developed to measure stressful life events that probably caused changes in the life of an adolescent a year before and a year after terrorist attack.
5. Beck Depression Inventory II for Children (BDI-II) (Beck et al., 1996). This scale consists of 21 items that measure depressive symptoms. Each item is answered on a 4-point response options ranging from 0 to 3. The scale has internal consistency Cronbach's α = .95. It has been widely used in clinical research and clinical practice (Gomes-Oliveira et al., 2012; Grothe et al., 2005).

Items measuring anxiety symptoms were adapted from a psychological distress scale by Kessler et al. (2002). The scale demonstrated high reliability coefficient of Cronbach's α = .79 and has been widely used among adolescents (Chan & Fung, 2014; Green et al., 2010; Huang et al., 2009). The study further included self-developed items on participants’ gender, religion, ethnicity, and school attendance.

### Procedure

Data collection took place between June and August 2021. Both children's verbal assent and informed consent from parents, caregivers and refugee camp managers were obtained before the respondents were asked to complete the questionnaire. For children whose parents had been killed, informed consent was obtained from National Emergency Management Authorities. The respondents were informed about the aims and objectives of the study. They were further informed that participation in the study was entirely voluntary, and they were allowed to opt out at any stage. To guarantee confidentiality of the information provided by the respondents, copies of the questionnaire retrieved from the respondents were not associated with the participant's name or any other means of identification. Ethical clearance was obtained from the University College Hospital Ibadan, before the study was conducted (UI/EC/21/0041).

The survey questionnaire was translated into the Hausa language by professional translators at the Department of Psychology, Federal University Gashua (FU Gashua). The questionnaire was back-translated into English to correct any errors that emerged from the first translation. Copies of the questionnaire were administered by two undergraduate students of FU Gashua. The two enumerators were natives of the community in which the study was conducted and were fluent in Hausa and the local language. Prior to data collection, the enumerators received 1 week of training on privacy, confidentiality, and scientific procedures of data collection. Respondents received compensation solely for their participation in the study. A total of 23 participants who showed marked psychological distress during assessment were referred to UNICEF's team and the health management team of the IDP camp for psychological counseling and therapy. The referral pathways began with the identification of distressed children by the enumerators, after which the IDP camp’s health management team was notified and facilitated referral to the UNICEF mental health and psychosocial support team. Detailed information about the procedures of data collection and ethical clearance can be found in the online Supplementary Material. Feedback on the findings of the study was conveyed to the participants through workshops and seminars.

### Data Analysis

The Statistical Package for Social Science version 29.0 was used for both descriptive and inferential statistics. The statistical models included chi-squared tests for independence, Pearson product moment correlation and multiple regression analysis for testing composite relationship of the independent variables. The hypotheses were supported or rejected at a *p* = .05 probability level.

## Results

A total of 391 children directly affected by Boko Haram terrorism were sampled in this study. The respondents were largely male (60.4%). Congruent with sampling, the age range was 11–23 years (*M*_age_ = 18.00, *SD* = 2.83). Ethnic group distribution was as follows: 129 Hausa (49.1%), 94 Kanuri (23.3%), 51 Bade (51(13.0%), 49 Fulani (12.5%), 5 Yoruba (1.3%) and 3 Igbo (0.8%). A total of 213 respondents (54.5%) said that both parents were alive, 149 (38.1%) reported that one parent had died, and 22 (5.6%) said that neither of their parents was alive.

Table 1 revealed that major life events as measured by the Adolescent Life-Change Events Scale (Yeaworth et al., 1992) significantly predicted psychosocial adjustment among the children (*R*^2^ = .364, *F*(6, 385) = 36.57, *p* < .01). The results further revealed that the independent contributions of life-change events involving family members (β = .231, *t* = 4.908, *p* < .051), close friend (β = .167, *t* = 3.466, *p* < .01), peers (β = .25, *t* = 4.75, *p* < .01), school (β = −.133, *t* = −2.71, *p* < .01) and health status events of the respondents (β = −.09, *t* = −2.07, *p* < .05) were significant predictors of psychosocial adjustment. Subscales of life-change events involving peers and experiences at school were associated with increased vulnerability to poor psychosocial adjustment. The results also revealed independent contributions of demographic variables, including ethnic affiliation (β = −.174, *t* = −3.25, *p* < .001), parental status (β = .159, *t* = 3.03, *p* < .013) and level of study (β = .130, *t* = 2.48, *p* < .01) to poor psychosocial adjustment. Loss of one or both parents increased the vulnerability of the participants to poor psychosocial adjustment (β = .159, *t* = 3.03, *p* < .013)

Table 2 shows a chi-squared test of independence that was conducted to examine the prevalence PTSD among children exposed to Boko Haram terrorism (no PTSD vs PTSD clinically presented symptoms as measured by the PTSD Checklist for DSM-5 by Ashbaugh et al., 2016). The result highlights the relationship between traumatic exposure/stressors and PTSD among children in terrorism-endemic areas. Based on the inclusion criterion of exposure to Boko Haram terrorism, all respondents had either mild (25.57%) or severe traumatic exposure (70.4%). Overall, 62.7% of the respondents reported clinically significant symptoms of PTSD with a PTSD diagnosis significantly more prevalent in respondents with severe traumatic exposure (*X*^2^(1) = 64.1, *p* = .003).

**Table 1.** Multiple Regression Analysis On Subscales of Life-Change Events and Demographic Variables On Psychosocial Adjustment.

		*B*				SE				*t*				*p*				95% Confidence Intervals			
Gender		−0.064				**.**779				−1**.**245				**.**214				[−2.502, 0.562]			
Age		0.029				.133				0.569				.570				[−0.187, 0.338]			
Religion		0.70				.784				1.366				.173				[−0.471, 2.614]			
Ethnicity		−0.174				**.**219				−3**.**242				**.**001				[−1.141, 0.279]			
Parental status		**0** **.** **159**				**.** **571**				**3** **.** **036**				**.** **003**				[**0.610**, **2.854**]			
Level of study		**0** **.** **130**				**.** **689**				**2** **.** **485**				**.** **013**				[**0.358**, **3.066**]			

Variable	Overall Psychosocial Effects				Thought and Risk Perception			Emotional Reaction			Circles of Vulnerability			Suspicion Distrust			Social Interaction			Freedom of Movement/Safety	
	*β*	*t*			*β*	*t*		*β*	*t*		*β*	*t*		*β*	*t*		*β*	*t*		*β*	*t*
Family events	.231	4.908**			.309	6.224**		.212	4.54**		.030	0.545		.079	1.441		−.001	−0.024		−.025	−0.462
Close friend events	.167	3.466**			.179	3.530**		.058	1.206		.051	0.896		.231	4.15**		.016	0.267		.005	0.085
Peers events	.252	4.751**			.143	2.565**		.406	7.71**		.169	2.69**		−.049	−0.797		.036	0.556		−.143	−2.38*
School events	.133	2.712**			.099	1.915		.034	0.706		.198	3.404**		.124	2.18*		−.038	−0.643		.034	0.604
Work non-school events	.035	0.727			−.048	−0.956		.000	0.010		−.089	−1.568		.087	1.550		.258	4.44**		−.073	−1.35
Health body events	−.09	−2.069*			−.116	−2.640**		−.194	−4.70**		.038	0.775		−.169	−3.49**		−.037	−0.735		.396	8.40**
*R*	.603				.542			.610			.323			.377			.262			.426	
*R* ^2^	.364				.294			.372			.104			.142			.069			.182	
*F*	36.57				26.62			37.96			7.44			10.63			4.709			14.20	
df	6, 385				6, 385			6, 385			6, 385			6, 385			6, 385			6, 385	
Significance	.001				.000			.000			.000			.000			.000			.000	

*p < .05, **p < .01, ***p < .001.

**Table 2.** Traumatic Exposure/Stressors and Post-Traumatic Stress Disorder (PTSD) Severity Symptoms.

Source		Traumatic Exposure								
		Mild		Severe		Total		Chi-Squared Tests		
		*N*	%	*N*	%	*N*	%	χ^2^	df	Significance
Post-traumatic stress	No PTSD	60	60.0	86	29.6	146	37.3	64.12	36	.003
	PTSD clinically presented	40	40.0	205	70.4	245	62.7	74.62	36	.001
Total		100	100	291 (74.42)	100	391	100	16.10	1	.001

As shown in Table 3, life-change events involving family, a close friend, peers, at school and work, or in non-school locations significantly and positively correlated with psychosocial adjustment, while life-change events involving health status of the respondents had a negative effect on psychosocial adjustment.

**Table 3.** Descriptive Statistics and Zero-Order Correlations for Study Variables.

	Variable	*M*	*SD*	1	2	3	4	5	6	7	8	9	10	11	12	13
1	Psychosocial adjustment	49.28	7.46		.605**	.764**	.585**	.554**	.433**	.067	.447**	.419**	.500**	.359**	.299**	−.072
2	Thought and risk perception	10.31	2.71			.411**	.169**	.249**	−.078	.148**	.457**	.380**	.376**	.251**	.176**	.124*
3	Emotional reaction	12.97	3.42				.326**	.261**	.165**	.197**	.419**	.329**	.533**	.281**	.226**	.196**
4	Circles of vulnerability	7.35	2.06					.273**	.148**	−.040	.137**	.160**	.268**	.269**	.096	.043
5	Suspicion distrust	5.74	1.81						.176**	.103*	.215**	.301**	.181**	.197**	.189**	.141**
6	Social interaction	7.45	2.08							.111*	.083	.107*	.129*	.090	.255**	.004
7	Freedom of movement/safety	5.46	1.61								−.113*	−.074	−.161**	−.037	−.055	.387**
8	Family	12.08	3.80									.428**	.409**	.209**	.268**	−.048
9	Close friends	13.05	3.67										.434**	.259**	.337**	.025
10	Peers	13.10	4.18											.505**	.412**	.010
11	School	12.98	4.18												.424**	.092
12	Work non-school	13.15	3.96													.171**
13	Health body	12.49	4.12													

*Correlation significant at the 0.05 level (two-tailed).

** Correlation significant at the 0.01 level (two-tailed).

To evaluate the psychosocial effects of exposure to Boko Haram terrorism, independent sample *t*-tests were conducted comparing children with high and low exposure. The results are given in Table 4. There was a significant effect of exposure to Boko Haram terrorism on psychosocial adjustment of the respondents (*t*(389) = 7.63, *p* < .001). The respondents were significantly different in their experiences of negative thought and risk perception (*t*(389) = 7.12, *p* < .001), emotional reaction (*t*(389) = 4.97, *p* < .001), circles of vulnerability (*t*(389) = 2.04, *p* < .05), suspicion and distrust (*t*(389) = 4.66, *p* < .001) and low social interaction (*t*(389) = 2.65, *p* < .008). The children reacted to Boko Haram terrorism in two major ways: emotional responses (e.g., aggression, fear, or uneasiness); and behavioral reactions (e.g., anxiety or sleep problems). The findings highlight that exposure to Boko Haram terrorism disrupts victims’ internal emotion regulation and external behavioral functioning.

**Table 4.** Psychosocial Effects of Exposure to Boko Haram Terrorism.

	Exposure	*N*	*Mean*	*SD*	*t*	df	*p*	Cohen's *d*
Psychosocial effects of terrorism	Low High	100 291	44.69 50.86	8.19 6.50	7.62	389	<.01	6.96951
Thought and risk perception	Low High	100 291	8.74 10.85	2.29 2.63	7.117	389	<.01	2.55192
Emotional reaction	Low High	100 291	11.55 13.46	3.12 3.39	4.969	389	<.01	3.32287
Circles of vulnerability	Low High	100 291	6.99 7.47	2.17 2.01	2.039	389	.042	2.04923
Suspicion distrust	Low High	100 291	6.99 7.47	2.17 2.01	2.039	389	<.02	2.04923
Low social interaction	Low High	100 291	6.98 7.61	2.36 1.95	2.645	389	<.008	2.06020
Lack of freedom of movement/safety	Low High	100 291	5.37 5.49	1.49 1.65	−0.632	389	.528	1.61135

Table 5 presents the frequencies and chi-square analyses of the PTSD symptoms with other mental health conditions. The finding shows that there was a significant proportion of children with PTSD who expressed anger (*X*^2^(1) = 5.81, *p* = .016) and depression (*X*^2^(1) = 3.95, *p* = .047). More than half (57.6%) of the children with PTSD reported aggression and 65.7% were depressed.

**Table 5.** Frequencies and Chi-Square for Co-Morbidity of Post-Traumatic Stress Disorder (PTSD) with Other Mental Illnesses.

		PTSD						Chi-squared Tests		
		No		Clinically presented		Total		χ^2^	df	Significance
		*N*	%	*N*	%	*N*	%			
Nervousness and problems sleeping	Never	68	46.6	104	42.4	172	44.0	1.69	1	.193
	Frequently experience	78	53.4	141	57.6	219	56.0			
		146	100	245	100	391	100			
Aggression	Never	67	45.9	96	39.2	163	41.7	5.812	1	.016
	Frequently experience	79	54.1	149	60.8	228	58.3			
		146	100	245	100	391	100			
Depression	Never	68	46.6	84	34.3	152	38.9	3.95	1	.047
	Frequency experience	78	53.4	161	65.7	239	61.1			
		146	100	245	100	391	100			
Anxiety	Never	52	35.6	84	34.3	136	34.8	.07	1	.789
	Frequency experience	94	64.4	161	65.7	255	65.2			

## Discussion

This study investigated the impact of Boko Haram terrorism on the psychosocial adjustment of children and young adults in northeast Nigeria. Data from a self-report survey revealed a high prevalence of clinically significant PTSD symptoms among children exposed to terrorism. Children's exposure to acts of Boko Haram terrorism was associated with both emotional responses (e.g., aggression, fear, uneasiness) and behavioral outcomes (e.g., anxiety, sleep disturbances), underscoring these associations without implying causation. Terrorism-induced distress further undermines children's perceptions of safety and coping mechanisms, which in turn contribute to lower school enrollment, particularly in a region with limited educational development.

Life-change events significantly influenced children's psychosocial adjustment. Respondents reported high levels of risk perception, heightened suspicion, and distrust. This result is consistent with Blum et al. (2014), Dohrenwend (2000) and Dupéré et al. (2018), who found that exposure to terrorism was associated with intense fear, heightened sense of insecurity, and elevated risk perception of future attacks. The findings are also supported by other studies (Keyes et al., 2014; Martin-Peña et al., 2011; Martin-Peña & Varela-Rey, 2014), which reported that exposure to terrorism undermined psychosocial functioning across the life course. In the case of northeastern Nigeria, a lack of social support, overstretched child protection mechanisms, inadequate human/material resources, and poor behavioral response skills are strongly associated with psychosocial maladjustment in children exposed to Boko Haram terrorism.

The adolescents reported high (70.4%) clinical symptoms of PTSD. This percentage is markedly higher than the global average of a 26% prevalence rate of clinical PTSD symptoms level in conflict settings (Hoppen & Morina, 2019; for individual studies, see Agadjaniana & Prata, 2003; Saiqa Razik et al., 2013). The percentage is also elevated compared with studies from other African conflict settings (Kim, 2019: Oskorouchi, 2019). Despite geographical and cultural differences, the findings of this study are consistent with past studies on terrorist events in France (Grenon et al., 2019), the Utoya Island terrorist attack in Norway (Dyb et al., 2014) and the attack on the Peshawar high school students in Pakistan, which reported elevated clinical symptoms of PTSD among survivors of the Utoya Island terrorist attack and among Peshawar high school students, respectively. PTSD prevalence rates in the current study also exceed rates reported in non-conflict settings, including Germany, where PTSD prevalence is typically below 20% (Wesemann et al., 2020).

It is imperative to note that terrorism-induced PTSD is particularly detrimental during adolescence, because this period represents a critical transition to adulthood during which individuals first develop a sense of security and identity. Thus, exposure to security threats and adversity at early stages of life could lead to impaired cognition which, in turn, may distort memory and learning outcomes (Cisler & Herringa, 2021; Malarbi et al., 2016).

Moreover, our results indicated that PTSD was often comorbid with other clinical and behavioral symptoms, such as depression, anxiety, and aggression. This implies that children who were exposed to terrorism are at risk not only of PTSD, but other mental health disorders as well (Betancourt et al., 2014; Dekel et al., 2014; Hansen et al., 2016; Horesh et al., 2017; Werner, 2012). These results are consistent with other survey studies (Dekel et al., 2017; Neria et al., 2011; Perlman et al., 2011; Rytwinski et al., 2013) and longitudinal studies (Caramanica et al., 2014; Flory & Yehuda, 2015; Ginzburg et al., 2010; Neria, 2011; Rytwinski et al., 2013) on the topic, which found a positive correlation between PTSD and depression among child victims 14 years after the 9/11 terrorist attacks in the United States. The finding is also in line with North et al. (1999) who found elevated levels of major depressive disorder among victims of terrorist attacks in Oklahoma City four months after the attack.

It is interesting to note that there is ongoing scholarly debate regarding the construct validity of a PTSD diagnosis among displaced adolescents. One of these arguments concerns the cultural validity of PTSD screening tools (de Fouchier et al., 2012; Kaltenbach et al., 2017; Magwood et al., 2022). Thus, establishing cross-cultural validity and age-appropriateness is critical for establishing the test accuracy of PTSD screening tools (Barbieri et al., 2019).

The current study also revealed that parental status, level of study and ethnic affiliation were associated with psychosocial adjustment. For example, loss of one or both parents also increased the vulnerability of the victims to psychosocial adjustment. This finding aligns with past studies (Burton et al., 2006) and affirms that loss of a primary caregiver is emotionally devastating and may trigger poor coping mechanisms (e.g., substance misuse, dissociation, emotional numbing). This finding is also supported by Melhem et al. (2011) who argue that children whose parents have been killed by terrorists are more likely to develop poor developmental, psychosocial, and behavioral health outcomes. Thus, loss of parents limits the potential career of a child because of the absence of parental figures to guide and monitor his/her career trajectories. Children in this category disproportionately bear the burden of Boko Haram terrorism and are at higher risk of being radicalized compared with children who have never experienced a terrorist attack or related violence.

The results demonstrate that approximately 20 million children were out of school because of insecurity and displacement (UNESCO Global Education Monitoring, 2023). This is in line with previous studies which found that direct exposure to terrorist attacks was associated with poor school attendance and poor social skills among Norwegian adolescents (Stene et al., 2019), and Peshawar students in Pakistan (Rodley et al., 2016). The result is also consistent with more recent evidence (Cabral et al., 2021) indicating that terror attacks in US high schools increase attrition rates and undermine children's chances of completing their studies. However, it is noteworthy that children's exposure to Boko Haram terrorism was extensive and persistent, unlike the case established in a nationally representative sample of Jerusalem adolescents (Bleich et al., 2003). Above all, our finding is consistent with Schuster et al. (2001) who reported that two-thirds of New York school-age children became terrified about their safety following the 9/11 attacks.

## Limitations of the Study

This study provides important insights into the effects of Boko Haram terrorism on children. It expands the scientific literature on terrorism-induced trauma among displaced children. However, there were some limitations. First, the survey was based on self-reported measures, which are subject to recall bias. Second, the study adopted a cross-sectional research design, making it impossible to establish a cause–effect relationship among the variables of the study. Third, data collection was carried out in terrorism-endemic northeast Nigeria, which posed a high security risk to the enumerators. This adversely affected the sample size and sampling validity. Lastly, we could not determine formal diagnostic criteria of PTSD because of an inability to conduct individual clinical interviews with the participants. Owing to these issues, findings are limited in their generalizability beyond the population of adolescents directly affected by Boko Haram terrorism in northeast Nigeria. Inability to fully culturally validate standardized tools in this study may affect the accuracy and generalizability of the findings. Future studies should prioritize cultural adaption of these tools. We were unable to assess participants’ cognitive and psychosocial maturity in relation to their responses to Boko Haram terrorism; this represents an important direction for future research.

## Conclusions

The psychosocial adjustment of children who have been directly exposed to terrorism is an important area of research worldwide, but little empirical evidence exists on this topic in sub-Saharan Africa, particularly in Nigeria. Therefore, this study addressed this gap in the literature. It was concluded that children who had direct exposure to Boko Haram terrorism reported elevated clinical symptoms of PTSD and had problems adjusting to social and educational life after attacks. Boko Haram terrorism-induced trauma has increased the number of out-of-school children and disrupts children's normal development and psychosocial functioning. The implication of this is that out-of-school children are particularly vulnerable to radicalization and forceful recruitment into terrorism. Besides, disruption in education adversely affects children's enrollment, and limits formal learning, thus undermining Nigeria's human capital development.

Understanding the effects of Boko Haram terrorism on mental health and psychosocial adjustment is crucial for special treatment needs of the victimized children. Trauma-focused cognitive-based therapy is particularly relevant, because it has the potential to mitigate the symptoms of mental health disorders. Children who have lost one or both parents to Boko Haram terrorism should be prioritized for intervention, given their heightened vulnerability to PTSD and poor psychosocial adjustment. In addition, victimized children can benefit from school-based interventions targeting emotion regulation skills and psychosocial supports.

Further studies should explore evidence-based interventions that could lower the odds of mental health problems of terrorist-exposed children. Longitudinal studies are needed to examine potential risk factors as well as long-term psychological effects of terrorist attacks on children's trajectories across lifespan. Also, clinical studies on evidence-based interventions are needed to provide useful information for effective treatment. Further studies should also consider using structured diagnostic interviews to obtain in-depth clinical assessment of the phenomenon. More importantly, child and neighborhood security and school safety should be given top priority and incorporated into the national security objectives of the government.

## Supplemental Material

sj-docx-1-tps-10.1177_13634615261436997 - Supplemental material for The Effects of Boko Haram Terrorism on Adolescents' and Young Adults' Psychosocial Adjustment in the Northeast of Nigeria Supplemental material, sj-docx-1-tps-10.1177_13634615261436997 for The Effects of Boko Haram Terrorism on Adolescents' and Young Adults' Psychosocial Adjustment in the Northeast of Nigeria by Wasiu Olorunlambe and Andreas Jud in Transcultural Psychiatry
