# Supplementary material for: The Effects of Boko Haram Terrorism on Adolescents' and Young Adults' Psychosocial Adjustment in the Northeast of Nigeria
Source: Transcult Psychiatry. 2026 May 13;63(3):219–33. doi: 10.1177/13634615261436997 (PMC13309627; doi:10.1177/13634615261436997)
Supplement: sj-docx-1-tps-10.1177_13634615261436997 - Supplemental material for The Effects of Boko Haram Terrorism on Adolescents' and Young Adults' Psychosocial Adjustment in the Northeast of Nigeria [file sj-docx-1-tps-10.1177_13634615261436997.docx]

**DEPARTMENT OF PSYCHOLOGY, FACULTY OF THE SOCIAL SCIENCES, UNIVERSITY OF IBADAN, IBADAN, NIGERIA**

Zuwaga kai/ke

**NEMAN YARDAR MASU AMSA TAMBAYOYI**

Wannan tambayoyin an tsarasurne kadai domin binkicen ilimi, sirrinka a ɓoye yake Kuma baza a bayyana shi ga kowa ba Sai don wannan binkicen. Babu buqatar suna kuma ba dole bane. Mungode da hadinkanka.

**SECTION A: ka/ki amsa wannan tambayoyin ta hanyar zaban daya a kowanne.**

1. Sashen A

1. Jinsi. Namiji ( ). Mace ( )

2. Shekaru............ ( Karubutq iya shekarun)

3. Addini musulunci. (. ). kiristanci ( ). Gargajiya ( )

4. Yare. Hausa ( ). Yoruba. ( ). Igbo ( ) wani daban............

5. Iyayenka suna Raye har yanzu? Duka na Raye ( ) daya naraye () duka sun mutu

6. Kana karatune yanzu? Idan Eh aji nawa Eh. ( ) a a ( )

7. Dawa kake rayuwa yanzu? Da mahaifiyata ( ) da mahaifina ( ) Uwa da uba( ) gurin Yan uwa ( ) gurin kula da yara/ sansanin hijira

**Sashen B: Yadda Sha anin Boko Haram yashafi mu amala**

| **S/No** | **Abubuwan** | **Ko kaɗan** | **wasu lokutan** | **koda yaushe** |
| --- | --- | --- | --- | --- |
| 1. | Inajin tsoron fita waje wasu lokutan |  |  |  |
| 2. | Naka damu akan abinda ka iya faruwa a gurin danake |  |  |  |
| 3. | Inajin tsoron tashin hankali |  |  |  |
| 4. | Iyalina ko ni kan gaji alokutan tashin hankali |  |  |  |
| 5. | Inada tinanin kaura daganan nakoma wani guri don gujewa tashin hankali |  |  |  |
| 6. | Idan naga ana kashe mutane nakanji nima wataran za a iya kasheni |  |  |  |
| 7. | Nakanji tsoron za a iya ganeni ko a nunani cikin jama a |  |  |  |
| 8. | Nakanji damuwa har nakasa barci saboda wannan matsalar |  |  |  |
| 9. | Wasu lokutan abin har Yana fin karfina |  |  |  |
| 10. | Saboda wannan matsalar nakan fada matuqar damuwa |  |  |  |
| 11. |  |  |  |  |
| 12. | Saboda wannan matsalar nakamu da cutar damuwa |  |  |  |
| 13. | Nakan damu sosai saboda wannan matsalar |  |  |  |
| 14. | Nakan shiga matuqar rashin jindadi da tsoro saboda wannan matsalar |  |  |  |
| 15. | Nakansha wasu magungunan don kaucewa damuwa |  |  |  |
| 16. | Wasu daga iyalina, abokaina, makusantan sungukeni |  |  |  |
| 17. | Wasu sungukeni har agurin aiki |  |  |  |
| 18. | Mu amalata da mutane ta ragu tundana shiga damuwa |  |  |  |
| 19. | Abokiyar zamana, yarana da sauran makusantan suma abin yashafesu har suna tsoron kada wani Abu yasameni |  |  |  |
| 20. | Nafara daina yarda da mutane |  |  |  |
| 21. | Nafara tunanin Ina kula danine |  |  |  |
| 22. | Nazama Mai yawan rashin yarda da yawan zargi saboda wannan matsalar |  |  |  |
| 23. | Ina shiga damuwa saboda kadaici Kuma mutane sun gujeni |  |  |  |
| 24. | Nakan killace kaina kokuma nisantar dakaina daga mutanen Dana Dani |  |  |  |
| 25. | Saboda wannan matsalar mutane na matuqar kyamata Kuma nakan kyamaci ayyukana nayau da kullum |  |  |  |
| 26. | An gujeni Kuma akan nunani a kauyenmu ko Garin mu |  |  |  |
| 27. | Nakan boye wannan yanayin ga Abokina aikina |  |  |  |
| 28. | Nadaina mu amala da wasu mutane |  |  |  |
| 29. | Mum sauya lokutan ayyukanmu nayau da kullum |  |  |  |
| 30. | Ina buqatar Jami in tsaro don tabbacin rayuwata |  |  |  |
| 31. | Bana samun damar ayyawatar da ayyukan yau da kullum |  |  |  |

**Shashen C: Ma aunin damuwa bayan afkuwar mummunan abu (PTSD Scale)**

**Instructions: waɗannan nadaga cikin abubuwan dake damun mutum bayan afkuwar mummunan abu.**

| S/No | A watan da ya gabata wane irin damuwa ka shiga | Kwata kwata | Nashiga kaɗan | Tsaka tsakiya | Nashiga sosai | Matuƙa |
| --- | --- | --- | --- | --- | --- | --- |
| 1. | Nakan yawaita tunanin abinda ya faru |  |  |  |  |  |
| 2. | Nakan yawaita mafarkin abubuwan dasuka faru |  |  |  |  |  |
| 3. | Nakanji kamar yanzu abin yake faruwa |  |  |  |  |  |
| 4. | Nakan shiga matuqar damuwa duk lokacin da wani abu ya tunomin abinda yafaru |  |  |  |  |  |
| 5. | Nakan afka mummunan yanayi duklokacin da natuna abinda yafaru. Misali, bugawar zuciya da suari, lumfashi dakyar, ko gumi |  |  |  |  |  |
| 6. | Ina kiyaye tunanin yanayin damuka shiga |  |  |  |  |  |
| 7. | Ina kiyaye abubuwan da zasu tinamin abinda yafaru misali, mutanen, guraren, ko firar abun |  |  |  |  |  |
| 8. | Ina da damuwar tunawa da muhimman al amurran da suka faru lokacin tashin hankali |  |  |  |  |  |
| 9. | Inada mummunan zato akan kaina, ko mutane koma duniya baki daya, misali, inajin kamar ni mutumin banzane, akwai mummunan abu dake damuwa, kowa ba a yarda bane, duniya duka mummunace |  |  |  |  |  |
| 10. | Ina ganin laifin kaina akan damuwar danake ciki ko abinda yafaru bayannnan |  |  |  |  |  |
| 11. | Inada babbar matsalar tsaro, tashin hankali dajin nike da alhakin wasu abubuwa |  |  |  |  |  |
| 12. | Nadaina jin dadin abubuwan da da kanyimin dadi |  |  |  |  |  |
| 13. | Nakanji kadaici ko nisanta kaina daga mutane |  |  |  |  |  |
| 14 | Inada matsalar fahimtar abubuwa masu kyau misali, bana gane farin ciki, ko nuna qauna ga makusantana |  |  |  |  |  |
| 15 | Halayyata batada tabbas, saurin fushi da daukan mataki cikin fushi |  |  |  |  |  |
| 16. | Nakanyi ganganci dayawa Wanda kan iya bani damuwa |  |  |  |  |  |
| 17. | Nakanjima ido biyu nakasa barci |  |  |  |  |  |
| 18 | Inada saurin rikicewa |  |  |  |  |  |
| 19 | Inada matsalar nutsuwa |  |  |  |  |  |
| 20 | Inasan wahala wajen yin barci ko kasancewa a barcin |  |  |  |  |  |

**Shashen D: Ma aunin damuwa lokacin da mummunan abu ke faruwa**

Ka nuna zurfin yadda ka yadda da waɗannan bayanan

| **S/No** | **Abubuwan** | **Kwatakwata ba gaskiya bane** | **Zai iya kasan cewa** | **Gaskiya ne** | **Tabbas gaskiyane** | **Gaskiya ne sosai** |
| --- | --- | --- | --- | --- | --- | --- |
| 1. | Nakanji ba mai taimakona |  |  |  |  |  |
| 2. | Na kanji damuwa da takaici |  |  |  |  |  |
| 3. | Nakan shiga damuwa da bakin ciki |  |  |  |  |  |
| 4. | Nakanji tsoro saboda tsaron lafiya ta |  |  |  |  |  |
| 5. | Nakanji nine da alhakin abun dake faruwa |  |  |  |  |  |
| 6. | Nakanji kunyar yadda nake abubuwa |  |  |  |  |  |
| 7. | Nakan damu sasboda tsaron lafiyar wasu |  |  |  |  |  |
| 8. | Inajin kamar bana cikin hayyacina |  |  |  |  |  |
| 9. | Inada matsalar rike kasha ko fitsari |  |  |  |  |  |
| 10. | Abunda nagani ya tayarmin da hankali matuqa |  |  |  |  |  |
| 11. | Inada bayyanannun damuwa kamar gumi ko karkarwa |  |  |  |  |  |
| 12. | Inajin kamar zan mutu |  |  |  |  |  |
| 13. | Ina tunanin zan iya mutuwa |  |  |  |  |  |

**Shashen E: Ma aunin rayuwar matashi:** Ka nuna zurfin yadda ka yadda da waɗannan bayanan

| **S/No** | **Abubuwan** | **Kwata kwata a a** | **Wasu lokutan** | **Ko dayaushe** | **Sosai da sosai** |
| --- | --- | --- | --- | --- | --- |
|  | **Abubuwan iyali** |  |  |  |  |
| 1. | Nakanyi gardama da iyalina |  |  |  |  |
| 2. | Bana gardamada iyalina |  |  |  |  |
| 3. | Nakan samu taimako daga iyalina duk lokacin dana ke buqaka |  |  |  |  |
| 4. | Bana samun taimakodaga iyalina in na nema |  |  |  |  |
| 5. | Ana barina nayi abinda nakesoo |  |  |  |  |
| 6. | Ba a barina nayi abinda nakeso |  |  |  |  |
|  | **Abubuwan makusantan abokai** |  |  |  |  |
| 7. | Nakanyi gardama da abokaina |  |  |  |  |
| 8. | Bana gardama da abokaina |  |  |  |  |
| 9. | Ina samun taimako daga abokaina in nanema |  |  |  |  |
| 10. | Bana samun taimako daga abokaina ko nanema |  |  |  |  |
| 11. | Abokaina sukan shiga muhimman al amurana |  |  |  |  |
| 12. | Abokaina basa shiga muhimman al amurana |  |  |  |  |
|  | **Abubuwan abokai** |  |  |  |  |
| 13. | Abokan karatu na kan addabata |  |  |  |  |
| 14. | Abokan karatuna na kareni |  |  |  |  |
| 15. | Ana gayyatata shagulan abokai |  |  |  |  |
| 16. | Akan wareni lokutan shagulan abokai |  |  |  |  |
| 17. | Nakansamun dadi lokucin kusanta |  |  |  |  |
| 18. | Bana samun dadi yayin kusanta |  |  |  |  |
|  | **Abubuwan makaranta** |  |  |  |  |
| 19. | Sakamakona a makaranta marar kyayune |  |  |  |  |
| 20. | Sakamakona na makaranta yanada kyau |  |  |  |  |
| 21. | Nakanyi ayyukana akan lokaci |  |  |  |  |
| 22. | Bana ayyukana akan lokaci |  |  |  |  |
| 23. | Malamina yana yabona akan ayyukana |  |  |  |  |
| 24. | Malamina baya yabona akan ayykana |  |  |  |  |
|  | **Abubuwan wurin aiki** |  |  |  |  |
| 25. | Nagane cewa zan iya abinda wasu baza su iyaba |  |  |  |  |
| 26. | Nagane cewa bazan iya abinda wasu suke ba\ |  |  |  |  |
| 27. | Nayi abinda nakanji alfahari akai |  |  |  |  |
| 28. | Ina alfahari da abinda nake |  |  |  |  |
| 29. | Nayi abinda a wajen makaranta an yabamin |  |  |  |  |
| 30. | Nayi abinda a wajen makaranta an kushe min |  |  |  |  |
|  | **Abubuwan lafiya da abokai** |  |  |  |  |
| 31. | Jikina yakoma daidai yadda nakeso |  |  |  |  |
| 32. | Jikina yakoma yadda bana so |  |  |  |  |
| 33. | Nakanyi rashin lafiya ko rauni saboda matsalar |  |  |  |  |
| 34. | Nasamu sauqi daga rashin lafiya nakuma warke |  |  |  |  |
| 35. | Wasu nazagina saboda yadda na kasance |  |  |  |  |
| 36. | Someone complimented me because of the way I look. |  |  |  |  |

**Table 6: Pattern of psychosocial effects of exposure to Boko haram terrorism by children**

|  | Item |  | Never | Often | Always |
| --- | --- | --- | --- | --- | --- |
| 1 | I have felt scared of going out at certain times and places | N | 263 | 89 | 39 |
|  |  | % | 67.3% | 22.8% | 10.0% |
| 2 | I have been worried about what might happen to the surroundings/ close environment. | N | 194 | 151 | 46 |
|  |  | % | 49.6% | 38.6% | 11.8% |
| 3 | I have felt afraid of an attack | N | 169 | 139 | 83 |
|  |  | % | 43.2% | 35.5% | 21.2% |
| 4 | My family or I have felt exhausted when facing this situation | N | 160 | 173 | 58 |
|  |  | % | 40.9% | 44.2% | 14.8% |
| 5 | I have thought about leaving everything and going to live somewhere else to put an end to the situation. | N | 139 | 162 | 90 |
|  |  | % | 35.5% | 41.4% | 23.0% |
| 6 | When you see that other people have been killed, you think it could also happen to you. | N | 170 | 128 | 93 |
|  |  | % | 43.5% | 32.7% | 23.8% |
| 7 | I have felt scared that I might be recognized or pointed out in some public places. | N | 147 | 122 | 122 |
|  |  | % | 37.6% | 31.2% | 31.2% |
| 8 | I have been nervous, and I have had problems sleeping as a result of the situation. | N | 151 | 125 | 115 |
|  |  | % | 38.6% | 32.0% | 29.4% |
| 9 | Sometimes I have felt overwhelmed by the situation. | N | 172 | 130 | 89 |
|  |  | % | 44.0% | 33.2% | 22.8% |
| 10 | As a result of the situation, I have felt outbursts of anger. | N | 170 | 139 | 82 |
|  |  | % | 43.5% | 35.5% | 21.0% |
| 11 | Faced with the situation, I have even become depressed. | N | 163 | 145 | 83 |
|  |  | % | 41.7% | 37.1% | 21.2% |
| 12 | I have felt a great deal of sadness about this situation. | N | 152 | 150 | 89 |
|  |  | % | 38.9% | 38.4% | 22.8% |
| 13 | I have suffered a great deal of uneasiness and anxiety because of the situation | N | 139 | 141 | 111 |
|  |  | % | 35.5% | 36.1% | 28.4% |
| 14 | I had to take some type of medication to be able to deal with the situation. | N | 136 | 142 | 113 |
|  |  | % | 34.8% | 36.3% | 28.9% |
| 15 | Some members of my family, my friends or acquaintances have distanced themselves from me. | N | 138 | 165 | 88 |
|  |  | % | 35.3% | 42.2% | 22.5% |
| 16 | Some people have distanced themselves from me | N | 144 | 164 | 83 |
|  |  | % | 36.8% | 41.9% | 21.2% |
| 17 | My family relations have deteriorated since I suffered this situation. | N | 163 | 138 | 90 |
|  |  | % | 41.7% | 35.3% | 23.0% |
| 18 | My immediate family members and other relatives have been affected by the situation I have experienced and are afraid that something might happen to me. | N | 158 | 145 | 88 |
|  |  | % | 40.4% | 37.1% | 22.5% |
| 19 | I began to distrust a lot of people. | N | 134 | 167 | 90 |
|  |  | % | 34.3% | 42.7% | 23.0% |
| 20 | I started to think that I was being observed. | N | 146 | 139 | 106 |
|  |  | % | 37.4% | 35.5% | 27.1% |
| 21 | I have become more distrustful and suspicious because of this situation. | N | 125 | 157 | 109 |
|  |  | % | 32.0% | 40.2% | 27.9% |
| 22 | I have felt afraid of being alone and people rejecting me. | N | 125 | 131 | 135 |
|  |  | % | 32.0% | 33.5% | 34.5% |
| 23 | I have isolated and distanced myself from the people I know. | N | 138 | 166 | 87 |
|  |  | % | 35.3% | 42.5% | 22.3% |
| 24 | As a result of the situation, I have felt a great deal of rejection and aversion to doing everyday things. | N | 149 | 152 | 90 |
|  |  | % | 38.1% | 38.9% | 23.0% |
| 25 | I have been marked or pointed out socially in my village or town. | N | 146 | 163 | 82 |
|  |  | % | 37.3% | 41.7% | 21.0% |
| 26 | I have had to hide this situation from friends | N | 143 | 146 | 102 |
|  |  | % | 36.6% | 37.3% | 26.1% |
| 27 | I avoided mixing with certain groups or people. | N | 169 | 159 | 63 |
|  |  | % | 43.2% | 40.7% | 16.1% |
| 28 | We have had to constantly change the times we do things, routes, routines and customs. | N | 245 | 124 | 22 |
|  |  | % | 62.7% | 31.7% | 5.6% |
| 29 | I needed an escort to guarantee my safety. | N | 127 | 223 | 41 |
|  |  | % | 32.5% | 57.0% | 10.5% |
| 30 | I have not felt free to carry out any everyday activity. | N | 147 | 117 | 127 |
|  |  | % | 37.6% | 29.9% | 32.5% |

Table 6: reveals that less than one-third (32.8%) felt scared of going out at certain times and places while 50.4% were worried about what might happen to the surroundings/ close environment. More than half (56.7%) of the respondents were afraid of terror attacks. Majority (54.4%) are nursing the thought about leaving everything and going to live somewhere else to put an end to the situation. More than half (56.5%) of the respondents on witnessing people being killed believed it could happen to them and many (64.2%) felt scared they could be pointed out to be killed in public places. The greater percentage (61.4%) have been nervous and had problems sleeping because of the situation. Most of the respondents reported being feeling overwhelmed (56.0%), expressed outbursts of anger (56.5%), depression (58.3%) great deal of sadness (61.0%), uneasiness and anxiety (64.5%). More than two-third (64.7%) reported that some members of their family, friends or acquaintances have distanced themselves from them and they also felt being distant from others (63.1%). 58.3% deteriorated family relations suffering.59.5% reported family members and other relatives affected by the situation experienced were afraid that something might happen to them. Close to two-third (62.6%) often or always felt they were being observed, 68.1% have become more distrustful and suspicious because of the situation as the majority (68.0%) felt afraid of being alone and people rejecting me. Two-thirds of the children (63.4%) often or always hid things from their friends, and more than half (56.1%) avoided mixing with certain groups.

Determine psychopathological comorbidity among children directly exposed to Boko haram terrorism. This question was analyzed using crosstab and chi-square test for independence. The result is presented in Table 7.


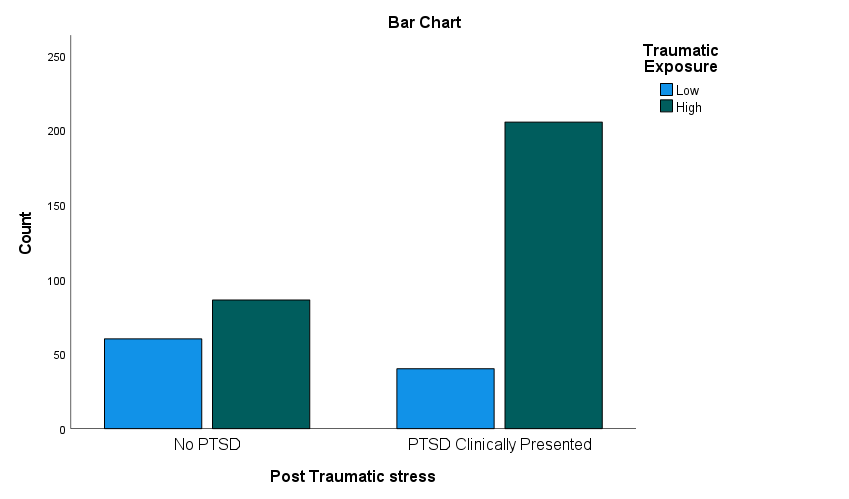


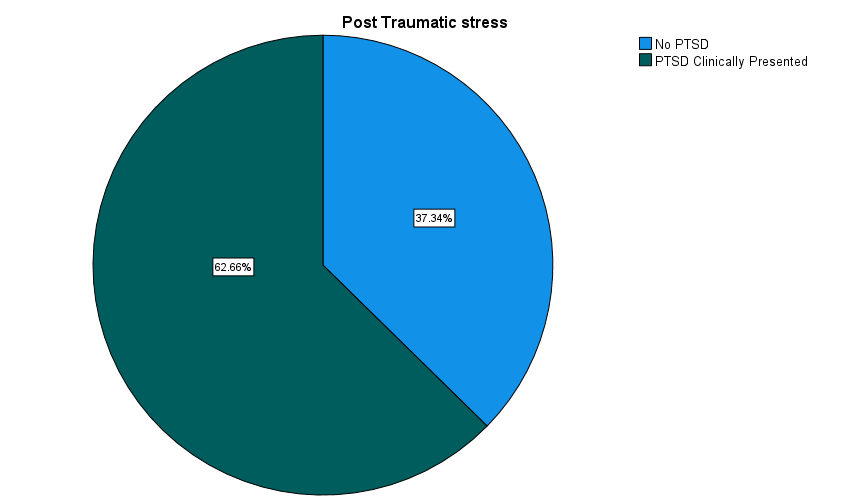


**Relationship between traumatic exposure and post-traumatic stress disorder**

**Table 4: Pattern of post-traumatic stress disorder profile sub-dimensions for youth**

|  | | Traumatic Exposure | | | | | | Total | | |
| --- | --- | --- | --- | --- | --- | --- | --- | --- | --- | --- |
|  |  | Mild | | | Severe | | |  | | |
|  |  | N | % | N | | % | N | | % |  |
| Post-Traumatic stress | No PTSD | 60 | 60.0% | 86 | | 29.6% | 146 | | 37.3% |  |
|  | PTSD Clinically Presented | 40 | 40.0% | 205 | | 70.4% | 245 | | 62.7% |  |
| Total | | 100 (25.57%) | 100.0% | 291 (74.42%) | | 100.0% | 391 | | 100.0% |  |

The pattern of Traumatic exposure revealed that 291 (74.42%) had severe traumatic exposure compared to 100(25.57%). Among those with severe traumatic exposure (291); 205 (70.4%) reported clinically significant symptoms of post-traumatic stress disorders compared to 40 (40.0%) who reported clinically significant symptoms of post-traumatic stress disorders from among those with mild exposure (100). Overall, 245(62.7%) of the respondents reported clinically significant symptoms of post-traumatic stress disorders.

Explore the influence of negative life events on psychosocial adjustment of children exposed to Boko Haram terrorism.

**Table 2: Mean Post-traumatic stress disorder among youths in terrorism endemic areas (n=391)**

|  | N | Minimum | Maximum | Mean | Std. Deviation |
| --- | --- | --- | --- | --- | --- |
| Posttraumatic stress | 391 | .00 | 66.00 | 34.3913 | 12.93070 |
| Valid N (listwise) | 391 |  |  |  |  |

The average scores on posttraumatic stress disorder were 34.39 (Standard deviation = 12.93). The scores range from 0 -66.

Figure 2: The bar chart showing the comparison between the proportions of PTSD occurrence and co-morbidity.

The bar chart demonstrates the proportion of children with high exposure to terror related activities who developed clinically significant post traumatic disorder (PTSD) and comorbidity of anger proneness and depression.

Table 5: Zero order correlations showing associations among the dimensions of psycho-social factors and life events.

| Variables | *M* | *SD* | 1 | 2 | 3 | 4 | 5 | 6 | 7 | 8 | 9 | 10 | 11 | 12 | 13 |
| --- | --- | --- | --- | --- | --- | --- | --- | --- | --- | --- | --- | --- | --- | --- | --- |
| Psycho-social adjustment | 49.28 | 7.46 | -- | .605^**^ | .764^**^ | .585^**^ | .554^**^ | .433^**^ | .067 | .447^**^ | .419^**^ | .500^**^ | .359^**^ | .299^**^ | -.072 |
| Thought and Risk perception | 10.31 | 2.71 |  | -- | .411^**^ | .169^**^ | .249^**^ | -.078 | -.148^**^ | .457^**^ | .380^**^ | .376^**^ | .251^**^ | .176^**^ | -.124^*^ |
| Emotional Reaction | 12.97 | 3.42 |  |  | -- | .326^**^ | .261^**^ | .165^**^ | -.197^**^ | .419^**^ | .329^**^ | .533^**^ | .281^**^ | .226^**^ | -.196^**^ |
| Circles of Vulnerability | 7.35 | 2.06 |  |  |  | -- | .273^**^ | .148^**^ | -.040 | .137^**^ | .160^**^ | .268^**^ | .269^**^ | .096 | .043 |
| Suspicion Distrust | 5.74 | 1.81 |  |  |  |  | -- | .176^**^ | -.103^*^ | .215^**^ | .301^**^ | .181^**^ | .197^**^ | .189^**^ | -.141^**^ |
| Social Interaction | 7.45 | 2.08 |  |  |  |  |  | -- | .111^*^ | .083 | .107^*^ | .129^*^ | .090 | .255^**^ | .004 |
| Freedom of Movement/Safety | 5.46 | 1.61 |  |  |  |  |  |  | -- | -.113^*^ | -.074 | -.161^**^ | -.037 | -.055 | .387^**^ |
| Family | 12.08 | 3.80 |  |  |  |  |  |  |  | -- | .428^**^ | .409^**^ | .209^**^ | .268^**^ | -.048 |
| Close friends | 13.05 | 3.67 |  |  |  |  |  |  |  |  | -- | .434^**^ | .259^**^ | .337^**^ | .025 |
| Peers | 13.10 | 4.18 |  |  |  |  |  |  |  |  |  | -- | .505^**^ | .412^**^ | .010 |
| School | 12.98 | 4.18 |  |  |  |  |  |  |  |  |  |  | -- | .424^**^ | .092 |
| Work Non-School | 13.15 | 3.96 |  |  |  |  |  |  |  |  |  |  |  | -- | .171^**^ |
| Health Body | 12.49 | 4.12 |  |  |  |  |  |  |  |  |  |  |  |  | -- |
| **. Correlation is significant at the 0.01 level (2-tailed). *. Correlation is significant at the 0.05 level (2-tailed). | | | | | | | | | | | | | | | |

Negative life events involving family, close friend, peers, at school and work or non-school locations significantly and positively correlated with psycho-social adjustment while negative life events involving the health status of the respondents was associated with poor psycho-social adjustment.

Children who have been affected by Boko haram terrorism will report high psychopathological comorbidity. Table 5: Wald statistics odd ratio children exposed to traumatic terror attacks who developed PTSD comorbidity

|  | *B* | *S.E.* | *Wald* | *df* | *Sig.* | *OR* | *95% C.I.for EXP(B)* | |
| --- | --- | --- | --- | --- | --- | --- | --- | --- |
| Depressed. | .532 | .235 | 5.116 | 1 | .024 | 1.703 | 1.074 | 2.700 |
| uneasiness and anxiety | -.174 | .239 | .528 | 1 | .467 | .841 | .526 | 1.343 |
| Anger outbursts. | .173 | .221 | .615 | 1 | .433 | 1.189 | .771 | 1.832 |
| Constant | -.318 | .497 | .409 | 1 | .522 | .728 |  |  |

The result reveals that the likelihood that participants with PTSD report other comorbidities. The logistic regression model was statistically significant, χ^2^(4) = 509.869^a^, p < .05. The model explained 2.4% (Nagelkerke R^2^) of the variance in depression and correctly classified 59.59% of cases PTSD.

**Chi-Square Tests**

|  | Value | Df | Asymptotic significance (2-sided) |
| --- | --- | --- | --- |
| Pearson Chi-Square | 64.162^a^ | 36 | .003 |
| Likelihood Ratio | 74.622 | 1 | <.001 |
| <.001 | 16.130 | 1 | <.001 |
| N of Valid cases | 391 |  |  |

1. 44 cells (59.5%) have expected count less than 5. The minimum expected count is .37.

Male participants expose to high trauma exposure will report a lower adjustment to terrorism compared to female participants who drive or do not are expose to high trauma exposure. The hypothesis was tested using 2x2 ANOVA and the result displayed in Table 1:

**Table 1:Two-way ANOVA showing the interaction between gender and trauma exposure on adjustment to terrorism among children.**

| Source | Type III Sum of Squares | df | Mean Square | F | Sig. |
| --- | --- | --- | --- | --- | --- |
| Gender | 12.026 | 1 | 12.026 | .249 | .618 |
| Exposure | 2524.149 | 1 | 2524.149 | 52.194 | <.001 |
| Gender * Exposure | 93.481 | 1 | 93.481 | 1.933 | .165 |
| Error | 18715.558 | 387 | 48.361 |  |  |
| Corrected Total | 21724.614 | 390 |  |  |  |

Table 1, shows that there was significant main effect of trauma exposure (F (1, 387) = 52.194, *p*<.01) on children’ adjustment to terrorism. However, there was no significant main effect of gender on adjustment to terrorism (F (1, 387) = .253, *p*>.05). The result also recorded that there was no significant interaction effect of trauma exposure and gender on adjustment to terrorism F (1, 387) = 1.93, *p*>.05).

**Table 4: Descriptive showing the mean differences in adjustment to terrorism based on trauma exposure**

|  | Mean | S.E |
| --- | --- | --- |
| Low | 44.756 | .707 |
| High | 50.688 | .418 |

The result of the mean differences in adjustment to terrorism shows that those with high trauma exposure reported more adjustment to terrorism compare to those with low trauma exposure.

**Table 4b: Descriptive showing the mean differences in adjustment to terrorism based on gender**

|  | Mean | S.E |
| --- | --- | --- |
| Male | 47.927 | .523 |
| Female | 47.517 | .633 |

The result of the mean differences in adjustment to terrorism shows that no significant gender difference adjustment to terrorism compare.

**Table 8: Descriptive showing the mean differences in adjustment to terrorism based on interactional dyads of trauma exposure and gender**

| Gender | Traumatic Exposure | Mean | Std. Error | LSD |  |
| --- | --- | --- | --- | --- | --- |
| Male | Low | 44.390 | .905 |  |  |
|  | High | 51.463 | .523 | -7.073^*^ |  |
| Female | Low | 45.122 | 1.086 |  |  |
|  | High | 49.912 | .651 | -4.790^*^ |  |

The result of the mean differences in adjustment to terrorism shows that males with high trauma exposure reported more adjustment to terrorism compare to males who had low trauma exposure and females with low and high exposure to trauma. Also, adjustment to terrorism was higher among those females with high trauma exposure compared to male and female respondents with low trauma exposure. However, the differences were not statistically significant. The hypothesis is thus rejected.


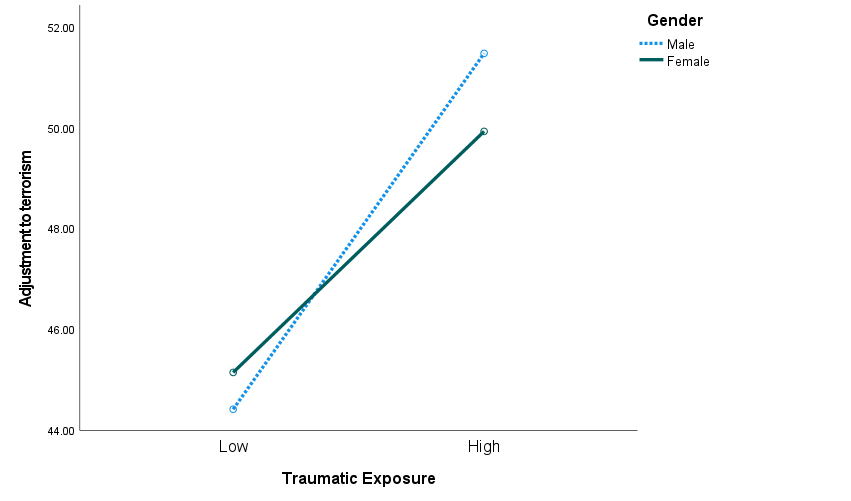


| **Tests of Between-Subjects Effects** | | | | | | |
| --- | --- | --- | --- | --- | --- | --- |
| Source | Dependent Variable | Type III Sum of Squares | df | Mean Square | F | Sig. |
| Gender | Thought and Risk perception | 10.614 | 1 | 10.614 | 1.647 | .200 |
|  | Emotional Reaction | 4.143 | 1 | 4.143 | .374 | .541 |
|  | Circles of Vulnerability | 11.760 | 1 | 11.760 | 2.807 | .095 |
|  | Suspicion Distrust | 2.557 | 1 | 2.557 | .824 | .364 |
|  | Social Interaction | 2.564 | 1 | 2.564 | .602 | .438 |
|  | Freedom of Movement/Safety | 27.594 | 1 | 27.594 | 10.947 | .001 |
| Exposure | Thought and Risk perception | 294.119 | 1 | 294.119 | 45.644 | <.001 |
|  | Emotional Reaction | 252.082 | 1 | 252.082 | 22.777 | <.001 |
|  | Circles of Vulnerability | 17.916 | 1 | 17.916 | 4.277 | .039 |
|  | Suspicion Distrust | 54.972 | 1 | 54.972 | 17.721 | <.001 |
|  | Social Interaction | 29.503 | 1 | 29.503 | 6.932 | .009 |
|  | Freedom of Movement/Safety | .018 | 1 | .018 | .007 | .932 |
| Gender * Exposure | Thought and Risk perception | 9.853 | 1 | 9.853 | 1.529 | .217 |
|  | Emotional Reaction | 2.229 | 1 | 2.229 | .201 | .654 |
|  | Circles of Vulnerability | .958 | 1 | .958 | .229 | .633 |
|  | Suspicion Distrust | 2.068 | 1 | 2.068 | .667 | .415 |
|  | Social Interaction | .070 | 1 | .070 | .016 | .898 |
|  | Freedom of Movement/Safety | 23.438 | 1 | 23.438 | 9.298 | .002 |
| Error | Thought and Risk perception | 2493.722 | 387 | 6.444 |  |  |
|  | Emotional Reaction | 4282.988 | 387 | 11.067 |  |  |
|  | Circles of Vulnerability | 1621.190 | 387 | 4.189 |  |  |
|  | Suspicion Distrust | 1200.494 | 387 | 3.102 |  |  |
|  | Social Interaction | 1647.115 | 387 | 4.256 |  |  |
|  | Freedom of Movement/Safety | 975.494 | 387 | 2.521 |  |  |
| Corrected Total | Thought and Risk perception | 2863.171 | 390 |  |  |  |
|  | Emotional Reaction | 4567.744 | 390 |  |  |  |
|  | Circles of Vulnerability | 1650.997 | 390 |  |  |  |
|  | Suspicion Distrust | 1271.391 | 390 |  |  |  |
|  | Social Interaction | 1680.777 | 390 |  |  |  |
|  | Freedom of Movement/Safety | 1011.054 | 390 |  |  |  |

Table 6, shows that there was no significant main effect of trauma exposure (F (1, 387) = 0.00, *p*>.05) on children’ Freedom of Movement/Safety. However, there was significant main effect of gender on Freedom of Movement/Safety (F (1, 387) = 10.95, *p*<0.01). The result also recorded that there was significant interaction effect of trauma exposure and gender on Freedom of Movement/Safety F (1, 387) = 9.30, *p*<.001).

**Table 6: Descriptive showing the mean differences in** Freedom of Movement/Safety **based on trauma exposure**

| Dependent Variable | Traumatic Exposure | Mean | Std. Error | 95% Confidence Interval | |
| --- | --- | --- | --- | --- | --- |
|  |  |  |  | Lower Bound | Upper Bound |
| Freedom of Movement/Safety | Low | 5.477 | .161 | 5.160 | 5.795 |
|  | High | 5.493 | .095 | 5.306 | 5.681 |

The result of the mean differences in adjustment to terrorism shows that there is no difference between respondents with high trauma exposure reported more Freedom of Movement/Safety compare to those with low trauma exposure.

**Table 7: Descriptive showing the mean differences in** Freedom of Movement/Safety **based on trauma exposure**

| Dependent Variable | Gender | Mean | Std. Error | 95% Confidence Interval | |
| --- | --- | --- | --- | --- | --- |
|  |  |  |  | Lower Bound | Upper Bound |
| Freedom of Movement/Safety | Male | 5.175 | .119 | 4.941 | 5.410 |
|  | Female | 5.795 | .145 | 5.511 | 6.080 |

The result of the mean differences in adjustment to terrorism shows that females reported more Freedom of Movement/Safety than males.

**Table 8: Descriptive showing the mean differences in** Freedom of Movement/Safety **based on interactional dyads of trauma exposure and gender**

| Gender | Traumatic Exposure | Mean | Std. Error | LSD | Sig. |
| --- | --- | --- | --- | --- | --- |
| Male | Low | 4.881 | .207 | 4.475 | 1.19* |
|  | High | 5.469 | .119 | 5.234 |  |
| Female | Low | 6.073 | .248 | 5.586 |  |
|  | High | 5.518 | .149 | 5.225 |  |

The result of the mean differences in Freedom of Movement/Safety shows that females with low trauma exposure reported more Freedom of Movement/Safety compare to females who had high trauma exposure and males with low and high exposure to trauma. Also, Freedom of Movement/Safety was higher among females with high trauma exposure compared to male and female respondents with low or high trauma exposure. The hypothesis is thus accepted.


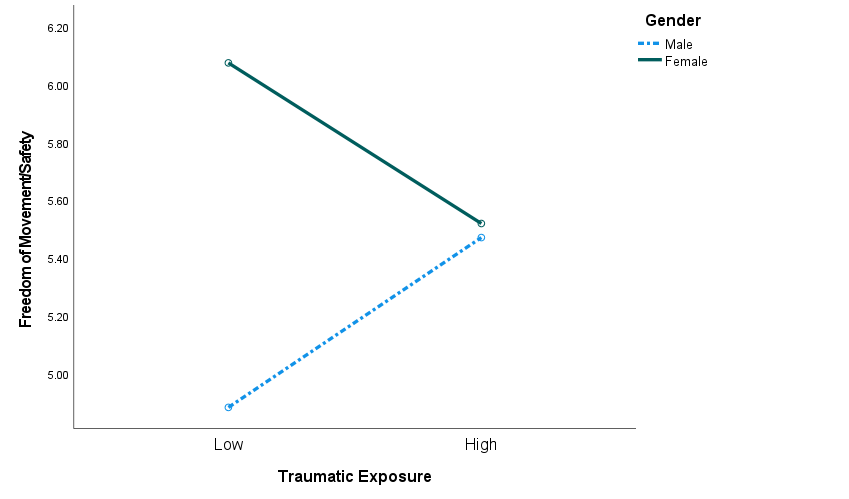


[DataSet2] /Users/wasiuolorunlambe/Downloads/BOKO HARAM ONILE WORK IV-7.sav

**Demographic variables**

| **Variables Entered/Removed^a^** | | | |
| --- | --- | --- | --- |
| Model | Variables Entered | Variables Removed | Method |
| 1 | Study, Ethnic, Age, Religion, Gender, Parent^b^ | . | Enter |
| a. Dependent Variable: Adjustment to terrorism | | | |
| b. All requested variables entered. | | | |

| **Model Summary^b^** | | | | |
| --- | --- | --- | --- | --- |
| Model | R | R Square | Adjusted R Square | Std. Error of the Estimate |
| 1 | .298^a^ | .089 | .074 | 7.18046 |
| a. Predictors: (Constant), Study, Ethnic, Age, Religion, Gender, Parent | | | | |
| b. Dependent Variable: Adjustment to terrorism | | | | |

| **ANOVA^a^** | | | | | | |
| --- | --- | --- | --- | --- | --- | --- |
| Model | | Sum of Squares | df | Mean Square | F | Sig. |
| 1 | Regression | 1925.979 | 6 | 320.997 | 6.226 | <.001^b^ |
|  | Residual | 19798.635 | 384 | 51.559 |  |  |
|  | Total | 21724.614 | 390 |  |  |  |
| a. Dependent Variable: Adjustment to terrorism | | | | | | |
| 1. Predictors: (Constant), Study, Ethnic, Age, Religion, Gender, Parent | | | | | | |

| **Coefficients^a^** | | | | | | | | |
| --- | --- | --- | --- | --- | --- | --- | --- | --- |
| Model | | Unstandardized Coefficients | | Standardized Coefficients | t | Sig. | 95.0% Confidence Interval for B | |
|  |  | B | Std. Error | Beta |  |  | Lower Bound | Upper Bound |
| 1 | (Constant) | 44.336 | 2.907 |  | 15.252 | <.001 | 38.621 | 50.052 |
|  | Gender | -.970 | .779 | -.064 | -1.245 | .214 | -2.502 | .562 |
|  | Age | .076 | .133 | .029 | .569 | .570 | -.187 | .338 |
|  | Religion | 1.071 | .784 | .070 | 1.366 | .173 | -.471 | 2.614 |
|  | Ethnic | -.710 | .219 | -.174 | -3.242 | .001 | -1.141 | -.279 |
|  | Parent | 1.732 | .571 | .159 | 3.036 | .003 | .610 | 2.854 |
|  | Study | 1.712 | .689 | .130 | 2.485 | .013 | .358 | 3.066 |
| a. Dependent Variable: Adjustment to terrorism | | | | | | | | |

| **Residuals Statistics^a^** | | | | | |
| --- | --- | --- | --- | --- | --- |
|  | Minimum | Maximum | Mean | Std. Deviation | N |
| Predicted Value | 43.9414 | 56.1419 | 49.2788 | 2.22225 | 391 |
| Residual | -22.27285 | 24.55585 | .00000 | 7.12501 | 391 |
| Std. Predicted Value | -2.402 | 3.088 | .000 | 1.000 | 391 |
| Std. Residual | -3.102 | 3.420 | .000 | .992 | 391 |
| a. Dependent Variable: Adjustment to terrorism | | | | | |

| **Residuals Statistics^a^** | | | | | |
| --- | --- | --- | --- | --- | --- |
|  | Minimum | Maximum | Mean | Std. Deviation | N |
| Predicted Value | 27.0000 | 73.0000 | 49.2788 | 7.46352 | 391 |
| Residual | .00000 | .00000 | .00000 | .00000 | 391 |
| Std. Predicted Value | -2.985 | 3.178 | .000 | 1.000 | 391 |
| Std. Residual | . | . | . | . | 0 |
| a. Dependent Variable: Adjustment to terrorism | | | | | |

**Life change events**

| **Variables Entered/Removed^a^** | | | |
| --- | --- | --- | --- |
| Model | Variables Entered | Variables Removed | Method |
| 1 | Health Body, Peers, Family, Work NonSchool, Close_friend, School^b^ | . | Enter |
| a. Dependent Variable: Adjustment to terrorism | | | |
| b. All requested variables entered. | | | |

| **Model Summary^b^** | | | | |
| --- | --- | --- | --- | --- |
| Model | R | R Square | Adjusted R Square | Std. Error of the Estimate |
| 1 | .603^a^ | .364 | .354 | 6.00026 |
| a. Predictors: (Constant), Health Body, Peers, Family, Work NonSchool, Close_friend, School | | | | |
| b. Dependent Variable: Adjustment to terrorism | | | | |

| **ANOVA^a^** | | | | | | |
| --- | --- | --- | --- | --- | --- | --- |
| Model | | Sum of Squares | df | Mean Square | F | Sig. |
| 1 | Regression | 7899.420 | 6 | 1316.570 | 36.568 | <.001^b^ |
|  | Residual | 13825.194 | 384 | 36.003 |  |  |
|  | Total | 21724.614 | 390 |  |  |  |
| a. Dependent Variable: Adjustment to terrorism | | | | | | |
| b. Predictors: (Constant), Health Body, Peers, Family, Work NonSchool, Close_friend, School | | | | | | |

| **Coefficients^a^** | | | | | | | | |
| --- | --- | --- | --- | --- | --- | --- | --- | --- |
| Model | | Unstandardized Coefficients | | Standardized Coefficients | t | Sig. | 95.0% Confidence Interval for B | |
|  |  | B | Std. Error | Beta |  |  | Lower Bound | Upper Bound |
| 1 | (Constant) | 31.473 | 1.679 |  | 18.745 | <.001 | 28.172 | 34.774 |
|  | Family | .454 | .093 | .231 | 4.908 | <.001 | .272 | .636 |
|  | Close_friend | .339 | .098 | .167 | 3.466 | <.001 | .147 | .531 |
|  | Peers | .450 | .095 | .252 | 4.751 | <.001 | .264 | .636 |
|  | School | .238 | .088 | .133 | 2.712 | .007 | .065 | .410 |
|  | Work NonSchool | .066 | .091 | .035 | .727 | .468 | -.112 | .244 |
|  | Health Body | -.156 | .075 | -.086 | -2.069 | .039 | -.304 | -.008 |
| a. Dependent Variable: Adjustment to terrorism | | | | | | | | |

| **Residuals Statistics^a^** | | | | | |
| --- | --- | --- | --- | --- | --- |
|  | Minimum | Maximum | Mean | Std. Deviation | N |
| Predicted Value | 40.3314 | 62.9771 | 49.2788 | 4.50055 | 391 |
| Residual | -25.42488 | 26.44436 | .00000 | 5.95392 | 391 |
| Std. Predicted Value | -1.988 | 3.044 | .000 | 1.000 | 391 |
| Std. Residual | -4.237 | 4.407 | .000 | .992 | 391 |
| a. Dependent Variable: Adjustment to terrorism | | | | | |

**Correlations**

[DataSet5]

| **Descriptive Statistics** | | | |
| --- | --- | --- | --- |
|  | Mean | Std. Deviation | N |
| Postraumatic stress | 34.3913 | 12.93070 | 391 |
| Peritraumatic stress | 20.4066 | 10.22294 | 391 |

| **Correlations** | | | |
| --- | --- | --- | --- |
|  | | Postraumatic stress | Peritraumatic stress |
| Postraumatic stress | Pearson Correlation | 1 | .356^**^ |
|  | Sig. (2-tailed) |  | <.001 |
|  | N | 391 | 391 |
| Peritraumatic stress | Pearson Correlation | .356^**^ | 1 |
|  | Sig. (2-tailed) | <.001 |  |
|  | N | 391 | 391 |
| **. Correlation is significant at the 0.01 level (2-tailed). | | | |

| **Confidence Intervals** | | | | |
| --- | --- | --- | --- | --- |
|  | Pearson Correlation | Sig. (2-tailed) | 95% Confidence Intervals (2-tailed)^a^ | |
|  |  |  | Lower | Upper |
| Postraumatic stress - Peritraumatic stress | .356 | <.001 | .266 | .440 |
| a. Estimation is based on Fisher's r-to-z transformation. | | | | |
